# Combining inertial navigation with transacetabular ligament in total hip arthroplasty via direct anterior approach results in excellent accuracy compared to standard manual technique: A retrospective cohort study

**DOI:** 10.1051/sicotj/2024013

**Published:** 2024-05-16

**Authors:** Vincent Maes, David Cossetto

**Affiliations:** 1 Royal North Shore Hospital, Department of Orthopaedic and Traumatic Surgery Reserve Rd, St Leonards NSW 2065 Sydney Australia; 2 University Hospitals Leuven, Department of Orthopaedic and Traumatic Surgery 49 Herestraat Leuven 3000 Belgium; 3 South Coast Orthopaedic Clinic 70 Bridge Road Nowra NSW 2541 Australia; 4 Nowra Private Hospital, Department of Orthopaedic Surgery Weeroona PI Nowra NSW 2541 Australia

**Keywords:** Total hip arthroplasty, Navigation, Transverse acetabular ligament

## Abstract

*Background*: Correct acetabular component placement plays a critical role in reducing early revisions after dislocations in total hip arthroplasty (THA). Although the transverse acetabular ligament (TAL) guides anteversion, inclination can only be accurately guided by navigation. In order to overcome the initial disadvantages with navigation, an imageless, easy-to-use inertial navigation system has been recently introduced. This study aims to analyze the accuracy of inclination with this navigation system compared to the standard manual technique. *Methods*: Two cohorts, manual technique (MT) and navigation (NAV) cohorts, consisted of 83 and 95 patients, respectively, after exclusion criteria were applied. Inclination target was 38° and anteversion was guided by TAL. Demographic data were collected, and anteroposterior (AP) pelvic and cross-table lateral radiographs were obtained 6 weeks post-operatively. Inclination and anteversion were determined on the AP pelvic and cross-table lateral radiograph, respectively. *Results*: A mean inclination of 41.8° (±6.8°) and 38.9° (±4.4°) was found in the MT and NAV cohorts, respectively. There was no statistical difference in gender, age, and BMI. If the inclination was set within 10° of the target (i.e., 38°), 88% of the MT cohort and 97% of the NAV cohort were within the target zone. Accuracy decreased to 53% and 83%, respectively, if the target zone range was narrowed down to ± 5°. *Conclusion*: Combining inertial imageless navigation for inclination and TAL as a landmark for anteversion is significantly more accurate compared to the manual technique, without having the limitations and disadvantages of current standard navigational techniques.

## Introduction

According to the Australian Orthopaedic Association National Joint Replacement Registry, 8.4% of total hip arthroplasty (THA) procedures are revised after 20 years, which makes it one of the most successful orthopaedic procedures to date [1]. However, despite the improvement in surgical techniques, implants, and technology, dislocation is still responsible for almost a quarter of the complications leading to early revision surgery [1]. Correct acetabular placement plays a crucial role in avoiding dislocation [2], early readmission, impingement, metallosis, and accelerated bearing wear [3]*.* A “safe zone” of 40° (±10°) of inclination (abduction angle) and 15° (±10°) of anteversion (version angle) was defined by Lewinnek et al. in 1978 [2]. Several other targets have been suggested since 1978, but accurately placing the acetabular component within the target zone remains a challenge [4, 5]. Callanan et al. reported 62% within their target of inclination and if combined with anteversion only 47% were within their target [4]. Furthermore, the femoral neck version and therefore combined anteversion could influence the acetabular “safe zone” as well [6].

In an attempt to overcome errors, computer navigation and robotics have been used. Although this led to a higher percentage of cups placed within the “safe zone” as well as a lower incidence of dislocation and subsequently revision [7, 8], the operation time became significantly longer and more expensive [9]. THA can also be done via the direct anterior approach (DAA) using intra-operative fluoroscopy [10]*.* However, there are some concerns regarding increased costs, operative time, accuracy, and radiation exposure. Another landmark for acetabular positioning is the transverse acetabular ligament (TAL) which can control acetabular height, depth, and anteversion but not inclination [11]. To date, there has been a low use of navigation in THA procedures [12]. Inertial imageless navigation devices were introduced to avoid some of these problems as they are easy to use, had no radiation exposure, and were sterile in use [13, 14]. This way, a patient can be located in a 3-dimensional space by just tilting the operating table (table tilt method), resulting in a functional baseline to the operating table (coronal plane). This functional coronal plane is similar to the reference plane used when measuring acetabular angles on a standard supine anteroposterior radiograph [14].

Our objectives were to look independently at the accuracy of inclination with the use of an inertial navigation device compared to the standard anatomical landmarks. Additionally, we looked at the influence of BMI and contralateral hip arthritis on this navigation tool. Finally, we were able to look at different anteversions of the TAL recorded via the navigation tool and compared these with the current literature of TAL orientation.

## Material and methods

### Study design

The study protocol of this retrospective cohort study adheres to the principles outlined in the declaration of Helsinki. This study was approved by the Northern Sydney Local Health District Human Research Ethics Committee (2023/ETH00062). This research did not receive any specific grant from funding agencies in the public, commercial, or not-for-profit sectors.

### Participants and settings

We retrospectively included all patients treated with a THA performed by the same surgeon (Author D.C.) via the DAA off-table from June 2020 until July 2022 at a single center in Nowra, New South Wales, Australia. All patients signed an informed consent for the use of de-identified data for research purpose. The following exclusion criteria were used: revision total hip arthroplasty, posterior approach, complications involving the cup (pelvic fracture, ceramic fracture), unavailability of the correct radiographs (AP pelvic and/or cross-table lateral hip radiograph), pelvic rotation on AP pelvis and radiographs performed at a different facility. Two cohorts (Manual technique (MT) and Navigation (NAV) cohorts) were established depending on the use of a standard manual or navigated technique of cup placement.

### Surgery and navigation

**Figure 1:**
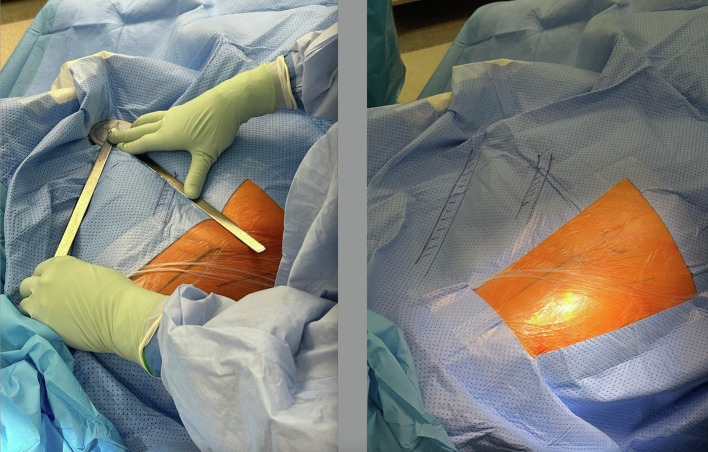
Drawing 38° with a goniometer on the drapes on a plane parallel to the operating table.

**Figure 2:**
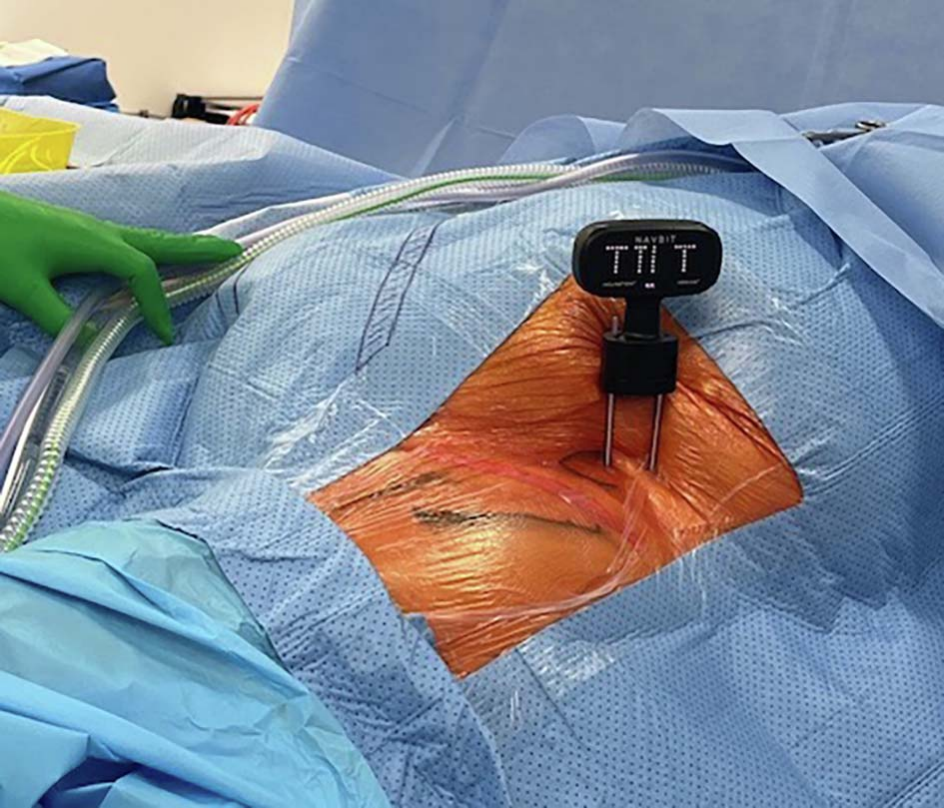
Set up inertial navigation device (Navbit^®^) during registration.

The patient was aligned with the operating table, the ipsilateral arm flexed over the chest and a contralateral thigh support was placed at the greater trochanter. The inclination target was set on 38° of inclination in all cases and anteversion was guided by the TAL. In the MT cohort, a goniometer was used to draw a line on the drapes to indicate 38° (Figure 1). As of the 27th of May 2021, the principal investigator consistently used an inertial imageless navigation device (Navbit^®^) (NAV cohort). The same technique of registration was used as previously described by Walter et al. [14] with one exception as the device was being used on the ipsilateral side instead of the contralateral side (Figure 2).

### Radiographs

A supine anteroposterior (AP) pelvis radiograph as well as a cross-table lateral radiograph were consistently performed at six weeks post-operatively at the same medical imaging center. The latter radiograph was carried out with the patient supine on the examination table and the contralateral hip flexed to 90°. The direction of the radiation beam was parallel to the examination table and 90° to the long axis of the body. The X-ray film was perpendicular to the examination table.

**Figure 3:**
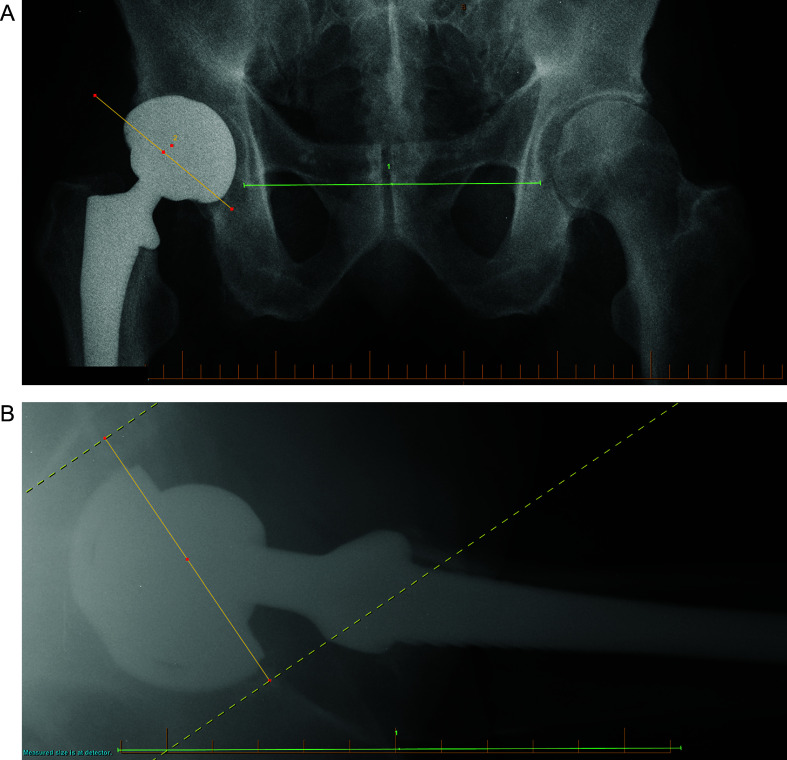
Acetabular cup inclination was determined as the angle (in degrees) between a line drawn along the rim of the cup and the teardrop line on the AP pelvis (A). Acetabular cup anteversion was measured on the cross-table lateral radiograph as the angle (in degrees) between a line drawn along the rim of the cup and a line perpendicular to the horizontal plane (B).

All measurements were systematically performed blinded by a hip and knee fellow (VM). First, the AP pelvis was reviewed for pelvic rotation or contralateral hip arthritis. Second, complications of the ipsilateral hip (fracture, cerclage wire) were noted. Third, acetabular cup inclination was determined as the angle (in degrees) between a line drawn along the rim of the cup and the teardrop line on the AP pelvis. Finally, acetabular cup anteversion was measured on the cross-table lateral radiograph, similar to the technique of Woo and Morrey [15]*.* It was determined as the angle (in degrees) between a line drawn along the rim of the cup and a line perpendicular to the horizontal plane (Figures 3A and 3B).

### Statistics

Multiple safe zone targets were used. We converted Lewennik’s original target zones of 40° inclination to 38° (±10°) inclination as we did have 38° as our main target and all measurements were performed in the coronal plane (AP pelvic X-ray) instead of the anterior pelvic plane (APP) in which they were originally defined. Callanan’s and Hevesi’s safe zone were already defined in the coronal plane. Hevesi’s safe zone was converted from 37° ± 10° to 38° ± 10°. Callanan’s safe zone was not converted as it had a similar target.

All basic statistics (e.g. student *t*-test, chi-square test) were performed in Microsoft^®^ Excel^®^. Statistically significant was defined as *p*-value < 0.05.

## Results

Two hundred and eleven patients were treated with a THA via DAA between June 2020 and July 2022. The MT cohort consisted of 95 patients and the NAV cohort contained 116 patients. After exclusion criteria were put in place, a total of 178 patients (181 hips) remained: 83(83) in the MT cohort and 95(98) in the NAV cohort (Table 1).

**Table 1:** Exclusion criteria, MT = Manual Technique cohort, NAV = Navigation cohort, unavailability of X-rays are subdivided in full and part of a set of X-rays.

	MT	NAV	Total
X-ray unavailable	8	16	24
Full	8	6	14
Part	0	10	10
AP pelvis rotated	3	5	8
Ceramic fracture	1	0	1
Total	12	21	33

Patient characteristics are displayed in Table 2. There were no statistically significant differences in gender (Chi-square test; *p* = 0.16), age (Student *t*-test; *p* = 0.25), and BMI (Student *t*-test; *p* = 0.58) between the two cohorts. There were no fractures or signs of loosening of the acetabular component in both cohorts.

**Table 2:** Patient characteristics and measurements. *N* = number of patients, *SD* = standard deviation, Min = minimum value, Max = maximum value.

	MT	NAV
*N* patients		
Initial	95	116
After exclusion	83	95
Gender		
Female	42	58
Male	41	37
Age		
Mean	70.83	69.18
*SD*	9.34	9.64
BMI		
Mean	29.5	29.1
*SD*	5.2	5.4
Min	20.8	17.6
Max	42.8	48.8
Measured cup inclination (°)		
Mean	41.8	38.9
*SD*	6.8	4.4
Min	4.8	28.0
Max	57.2	55.5

**Figure 4:**
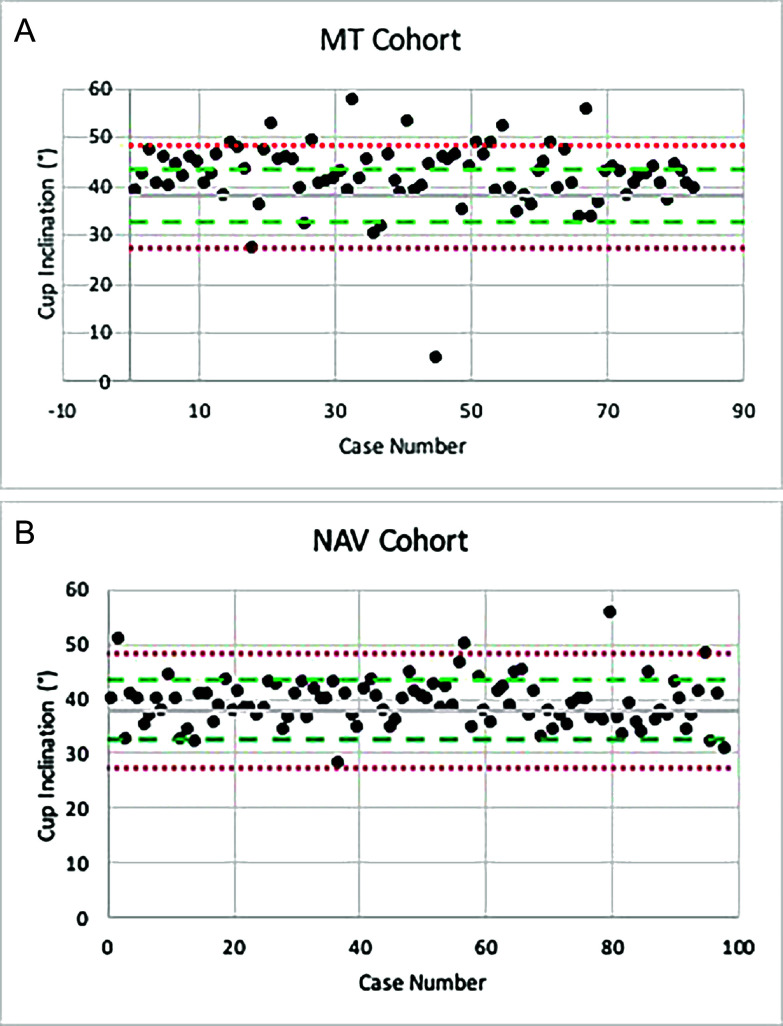
Cup inclination in degrees per case. (A) and (B) represent the MT and NAV cohorts, respectively. Grey line represents the target (38°). The dotted red line is within 10° of target and the dotted green lines represent within 5°.

There was a mean inclination of 41.8° (*SD* = 6.8°) in the MT cohort. The NAV cohort had a mean inclination of 38.9° (*SD* = 4.4°) (Table 2). Three different target zones were evaluated: within 10° (Lewinnek et al. [2]; Hevesi et al*.* [16]), within 30°–45° (Callanan et al. [4]), and within 5° of the target (38°) (Table 3). The NAV cohort was significantly more accurate independent of which target zone was chosen (Table 3). All inclinations of both cohorts can be viewed in Figures 4A and 4A.

**Table 3:** Percentage of cup inclination measurements within three different target zones.

	MT	NAV	Chi-square test
Lewinnek et al. (within 10°: 28–48°)	88.0%	96.9%	*p* = 0.02
Callanan et al. (30–45°)	71.1%	93.9%	*p* < 0.001
Within 5° (33–43°)	53.0%	82.7%	*p* < 0.001

As cup anteversion was dictated according to the TAL in both cohorts, our navigation tool measured the orientation. A mean anteversion of 25.8° ± 3.1° was measured.

An inclination error of −0.7° ± 5.4° was found, defined as the difference between the navigation measurement and the measured radiographic inclination in degrees.

**Figure 5:**
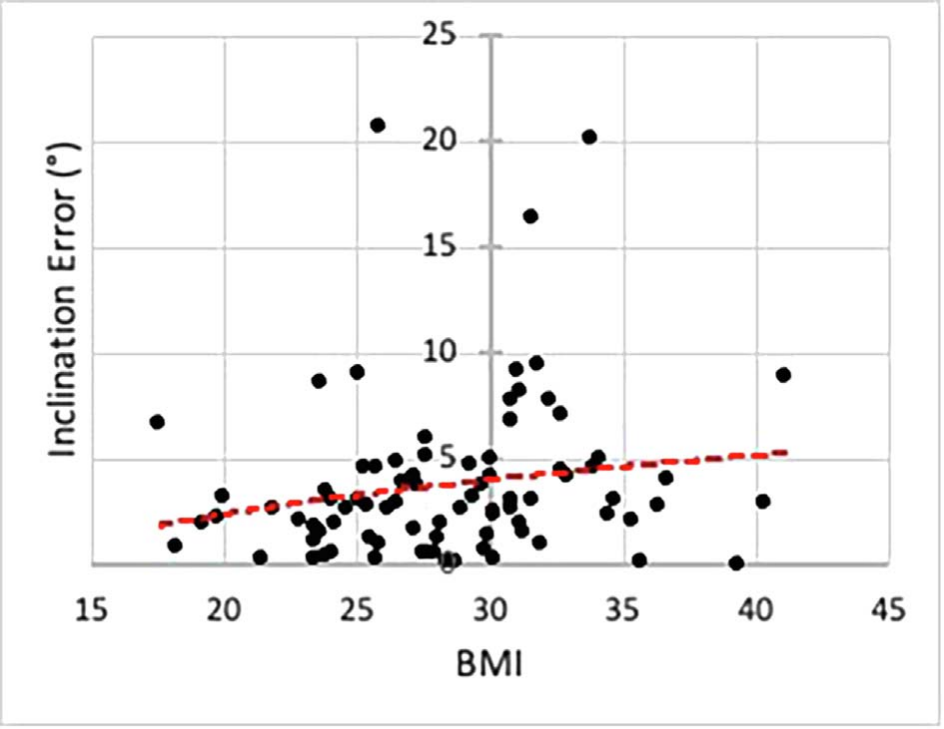
The inclination error is defined as the difference between the navigation measurement and the measured radiographic inclination in degrees. This is plotted in accordance with BMI. Red dotted line represents a trendline.

The influence of BMI on the inclination error is presented in Figure 5. A student *t*-test was performed using BMI 25 kg/m^2^ as the cutoff between the two groups. A *p*-value of 0.06 showed no significant difference. However, when the limit was raised to BMI 30 kg/m^2^, a significant difference (*p*-value 0.02) was found. The trendline suggests an average error of less than 5° with up to a BMI of 30 kg/m^2^, and just above 5° with a BMI of 30 kg/m^2^ or greater. There was no influence of contralateral hip osteoarthritis on the measured error (*p*-value 0.86). Finally, there were no adverse events (i.e., infection, fracture, and nerve injury) with Navbit^®^ except for two malfunctions during registration for which they needed to be replaced.

## Discussion

Accuracy of acetabular component placement is significantly greater when combining an inertial imageless navigation device with TAL, independent of patient age and contralateral hip arthritis. When using a conventional manual instrumentation, significant influence of BMI has been reported [4]. In our study, however, we only found a significant influence of BMI > 30 kg/m^2^ on inclination accuracy. This improvement in accuracy has the potential to avoid dislocation [2], early readmission, impingement, metallosis, and accelerated bearing wear [3].

Accuracy of inclination was also significantly better independent of the chosen safe zones (target zone). Lewinnek et al. originally described a safe zone for inclination (40° ± 10°) and anteversion (15° ± 10°) in the APP [2]. Callanan et al*.* narrowed the inclination safe zone down to 30°–45° in the coronal plane, based on surgeon consensus and standards from previous studies [4]. A fairly new inclination safe zone of 37° (±10°) in the coronal plane, based on the lowest dislocation hazards in a multivariable model, was defined by Hevesi et al. in 2022 [5]. Comparison of these safe zones is complicated as they each are defined within a specific different reference plane. In order to use the conversion formula proposed by Murray et al. and Zhinian Wan et al. to compare these safe zones, one needs the patient-specific pelvic tilt [17, 18]. Only when the pelvic tilt is 0°, the APP and the (radiographic) coronal plane are parallel and comparable. A wide variation of the APP has been described to the coronal plane with a mean supine pelvic tilt of 4.2°, ranging from −20.5° to 24.5° [19]. Also, the surgeon needs to be aware of different standing versus supine targets as the pelvis will tilt 5.5° posteriorly on average [19]. Therefore, we can only conclude that it is almost impossible as well as incorrect to directly compare literature results based entirely on the safe zone. A specific acetabular component orientation may be in the safe zone by one definition but outside the safe zone by other definitions. We therefore looked at every safe zone independently and found a significant improvement of inclination accuracy in all of them.

TAL was used as guidance for anteversion. The TAL is defined as a bridge across the inferior acetabular notch, continuing the outer edge of the acetabulum and is identifiable in 99.7% [20]. Unrelated to the patient’s position, TAL is known to be a reliable landmark for acetabular height, depth, and version. Unfortunately, TAL is unable to define acetabular inclination [11]. Multiple computer tomography (CT) studies assessed the native anatomy of TAL orientation in over 100 hips each and they consistently reported anteversion values of >15° [21]. This is comparable to our results where a mean anteversion of 25.8° to the coronal plane was measured via the inertial navigation device. Moskal et al. [22] already suggested the ideal combination of patient-specific morphology (PSM) (e.g. TAL) and computer-assisted navigation (CAN) for optimal acetabular component placement. This combination has the potential to be both reliable and accurate while avoiding the potential flaws of both techniques separately [22].

Although computer navigation and robotics were introduced in order to overcome accuracy, errors of manual (conventional) instrumentation, longer operation time, and increasing cost led to a low use in THA [12]. Recently, Kunze et al. [9] performed a systematic review of 12 RCT and found that manual THA resulted in 8.6 min and 23.4 min shorter procedure compared with computer-navigated and robotic THA, respectively. The mean additional time reported for this inertial imageless navigation device (Navbit^®^) was 6.73 min [14], which is comparable to others.

Regarding adverse events, there was a trend toward increased incidence of all-cause complications with manual THA compared with navigation-based THA, although no significant difference was shown [23]. A similar trend was found in a large database of 803,732 THA, where navigation-based THA was associated with reductions in complications as well as lower readmission rates compared to manual THA [7]. This is probably due to a markedly better acetabular cup accuracy of navigation-based THA when compared with manual THA. Kunze et al. found that more cups (79%) were placed within the Lewinnek safe zone with navigation-based THA compared to manual THA (52%) [9]. Our results have the same trend (88% manual and 97% navigation), though are relatively higher. This could be due to the experience of the surgeon and/or the measurement technique of the radiograph. Xu et al. recently confirmed the reliability of this inertial navigation tool compared to optical navigation and concluded that both navigation systems will allow for adequate acetabular positioning [24].

This study has several limitations. First, the significant increase in accuracy is not linked to clinical data on dislocations and patient outcomes. However, it has been previously shown that correct acetabular placement plays a crucial role in avoiding dislocation [2]. Second, the number of patients included in this study is rather limited. Third, measurements were performed on plain radiographs. Although concerns regarding accuracy and repeatability have been made, radiographs are easily accessible, inexpensive, widely used, and could be performed supine and/or standing. On the other hand, CT’s have a relatively low availability, increasing costs, and ionizing radiation exposure and can only be done in a supine position. Fourth, different measuring techniques have different accuracies. Bayraktar et al. compared the accuracy of cup orientation measurements between an AP pelvic radiograph and 3D-CT [25]. A mean absolute difference of 3.1° was found for inclination using the inter-teardrop line. Anteversion measurements, on the other hand, were more susceptible to errors with mean inaccuracies over 7° [25]. Finally, different methods have been described to measure anteversion on either an AP pelvis or cross-table lateral radiograph. The latter is described by Woo and Morrey [15]. They already recognized that the measured anteversion does not reflect true anteversion but more apparent anteversion. When using this measuring method, variations in positioning of the patient (rotation and/or pelvic tilt) may lead to inaccurate measurements of cup orientation [15]. Ghelman et al. already pointed out that notable differences exist between the CT and cross-table lateral radiograph measurements of version, as a difference of ≥ 10° was detected in 28% [26]. These variations in measurement could partly explain the difference in the proposed anteversion “safe zone of 28° (±10°)” defined by Hevesi et al. [5]*.*

In order to limit this measurement bias in our study, anteversion was described as apparent anteversion and not true anteversion. Furthermore, all measurements were done by one fellow in a short amount of time and two cohorts with similar demographics were established to minimize this measurement bias.

## Conclusion

Combining inertial imageless navigation for inclination and TAL as landmark for anteversion leads to a significantly better accuracy in acetabular cup placement without having the limitations and disadvantages of current standard navigational techniques. This technique achieves significantly higher accuracy independent of every different “safe zone” definition. Future studies should investigate clinical outcomes as well as cost effectiveness of combining computer navigation with patient-specific anatomic landmarks.

## Data Availability

The data is de-identified stored at the Tom Reeve Academic Surgical Clinic, Kolling Institute (Sydney, Australia). The study site at the NSLHD (Tom ReeveAcademic Surgical Clinic) has been ethically approved for the storage and application of identifiable patient data.
